# First Evidence of West Nile Virus Overwintering in Mosquitoes in Germany

**DOI:** 10.3390/v13122463

**Published:** 2021-12-09

**Authors:** Helge Kampen, Birke Andrea Tews, Doreen Werner

**Affiliations:** 1 Friedrich-Loeffler-Institut, Federal Research Institute for Animal Health, Institute of Infectology, 17493 Greifswald-Insel Riems, Germany; birke.tews@fli.de; 2 Leibniz-Centre for Agricultural Landscape Research, 15374 Muencheberg, Germany; doreen.werner@zalf.de

**Keywords:** *Culex pipiens*, first report, Germany, mosquito vectors, overwintering, transmission, West Nile virus

## Abstract

Mosquitoes collected from mid-December 2020 to early March 2021 from hibernacula in northeastern Germany, a region of West Nile virus (WNV) activity since 2018, were examined for WNV-RNA. Among the 6101 mosquitoes tested in 722 pools of up to 12 specimens, one pool of 10 *Culex pipiens* complex mosquitoes collected in early March 2021 in the cellar of a medieval castle in Rosslau, federal state of Saxony-Anhalt, tested positive. Subsequent mosquito DNA analysis produced *Culex pipiens* biotype *pipiens*. The pool homogenate remaining after nucleic acid extraction failed to grow the virus on Vero and C6/36 cells. Sequencing of the viral *NS2B-NS3* coding region, however, demonstrated high homology with virus strains previously collected in Germany, e.g., from humans, birds, and mosquitoes, which have been designated the East German WNV clade. The finding confirms the expectation that WNV can overwinter in mosquitoes in Germany, facilitating an early start to the natural transmission season in the subsequent year. On the other hand, the calculated low infection prevalence of 0.016–0.20%, depending on whether one or twelve of the mosquitoes in the positive pool was/were infected, indicates a slow epidemic progress and mirrors the still-hypoendemic situation in Germany. In any case, local overwintering of the virus in mosquitoes suggests its long-term persistence and an enduring public health issue.

## 1. Introduction

West Nile virus (WNV) is a flavivirus (family Flaviviridae) pathogenic to humans, horses, and birds. While the majority of infections in humans are asymptomatic and only a few infected people develop neurological symptoms, the virus is quite regularly neuroinvasive in horses and certain groups of birds, causing a high degree of mortality [1]. WNV is transmitted by mosquito species of various genera, with *Culex pipiens* being the most important vector species [2]. Susceptible birds not prone to developing the disease (mainly Passeriformes) act as virus vehicles, amplifiers, and reservoirs, infecting feeding mosquitoes, which may then transmit the virus to other groups of vertebrates [3,4]. Horses and humans are considered viral dead-end hosts, which do not develop viremias sufficiently high to infect feeding mosquitoes [5].

During the large European West Nile virus epidemic in 2018, which affected 15 countries with 2083 human infections, including 166 fatal ones, and 285 equine outbreaks in EU member states [6,7], the virus emerged for the first time in Germany [8]. In both 2019 and 2020, case numbers increased in Germany, with the first human case diagnosed in 2019 and the first fatal human case occurring in 2020 [9,10]. Confirmed cases were determined in northeastern Germany, notably in areas of Berlin and Leipzig [11].

In 2019, field-collected mosquitoes in Germany were found to be infected with WNV for the first time. In the Berlin Game Park where birds had previously succumbed to WNV infection, mosquito collections and viral screening produced seven WNV-positive pools of *Cx. pipiens* biotype *pipiens* and *Cx. pipiens* biotype *pipiens/molestus* mosquitoes [12].

The continuation of the epidemic over the years raised the question whether the virus was regionally maintained during wintertime or was repeatedly introduced by birds during the summer. Phylogenetic comparisons of WNV strains isolated in Germany and other European regions in different years and from different hosts showed a close relationship among most of the German strains, and suggested an introduction from the neighboring Czech Republic [8,9]. Repeated seasonal reintroduction from other European countries can therefore not be discounted. On the other hand, viral maintenance in overwintering female mosquitoes has been demonstrated elsewhere [13,14,15,16,17], suggesting that reintroduction is not necessary to start a new transmission season. To check for WNV overwintering in mosquitoes in Germany, hibernating adult mosquitoes were collected from their resting places during the winter of 2020/2021 in northeastern Germany, the known hotspot region of WNV emergence.

## 2. Materials and Methods

### 2.1. Mosquito Collection and Identification

Mosquitoes were collected between mid-December 2020 and mid-March 2021 in 20 bat hibernation quarters (ice cellars, tunnels, bunkers, water regulation constructions, and underground levels of abandoned factories) in the German federal states of Berlin, Brandenburg, Saxony, and Saxony-Anhalt, access to which was obtained through bat specialists counting overwintering bats. Mosquitoes were manually collected with vials from their resting sites on walls and ceilings, put into a transportable freezing box (−20 °C) to kill and fix them, and brought into the laboratory, where the vials were put into the freezer (−20 °C) until further processing. Mosquito identification took place morphologically to species or complex/group level on a chilling table under a stereomicroscope, using determination keys by Schaffner et al. [18] and Becker et al. [19]. For RNA/DNA extraction, mosquitoes were then pooled according to species, and collection site and date, with up to five specimens in the case of *Culiseta* species and up to 12 specimens in the case of *Culex* and *Anopheles* species.

For both viral screening and genetic mosquito identification, nucleic acid extraction was performed following Kampen et al. [12]. Genetic identification of mosquitoes in the *Cx. pipiens* complex pool was conducted according to Heym et al. [20].

### 2.2. Viral Screening and Characterisation

Extracts from the mosquito pools were subjected to a Flaviviridae real-time PCR targeting the *NS5* gene [21] and a Pan-Alphaviridae-PCR targeting the *nsP4* gene [22]. A pool positive for Flavivirus-RNA was cross-checked by unidirectional sequencing of the PCR product using the forward primer and the BigDye Terminator v1.1 Cycle Sequencing Kit (Applied Biosystems/Hitachi, Darmstadt, Germany).

### 2.3. Cell Culture and Virus Isolation Attempt

Vero cells (L0015, Collection of Cell Lines in Veterinary Medicine, Friedrich-Loeffler-Institut, Greifswald, Germany) are routinely grown in MEM with Hank’s and Earle’s salts, non-essential amino acids, and 10% fetal bovine serum (FBS) at 37 °C and 5% CO_2_. C6/36 cells are kept in Schneider’s insect medium supplemented with 10% FBS at 28 °C.

Virus isolation was attempted as described in Kampen et al. [12]. Briefly, the mosquito homogenate was applied to Vero and C6/36 cells in the presence of penicillin and streptomycin (Gibco/Thermo Fisher Scientific, Dreieich, Germany) and cultivated for several days, with daily observation to check for the appearance of a cytopathic effect.

Cells were passaged once and co-cultured with naïve cells during the cultivation period. Cells and cell culture supernatant were tested for the presence of viral RNA by two RT-qPCRs, using primers targeting either the 5′-untranslated region or the NS2A region [23]. Samples were considered positive with Ct values higher than that expected simply through the addition of the inoculum.

### 2.4. RNA Extraction and Partial Sequencing of the Viral NS2B-NS3 Region

Fresh RNA was isolated for genomic sequencing of the positive sample: mosquito homogenate supernatant (140 µL) was extracted using a QIAamp Viral RNA Mini Kit (Qiagen, Hilden, Germany) according to the manufacturer’s instructions. The *NS2B-NS3* coding region was partially amplified using a two-step RT-PCR approach. cDNA was generated using primer R11029 (GATCCTGTGTTCTCGCACCACCAG [24]) and ProtoScript (New England Biolabs, Ipswich, MA, USA) according to the manufacturer’s instructions. We added 2 µL of the cDNA into a PCR using KOD-DNA polymerase (Merck, Darmstadt, Germany) and in-house primers BT1232 (TGCGGACACCGTGGACCTGC) and BT1233 (GTGGCATGGCACATGACATCAAC). The resulting 2066 bp amplicon was sequenced with BT1233, and the sequence obtained was compared to sequences found in German mosquitoes collected in 2019 (GenBank accession nos. LR743447 and LR743455 [9]) using Geneious Prime Software version 2019.2.3 (Geneious Biomatters, Auckland, New Zealand).

## 3. Results

### 3.1. Mosquito Collections

A total of 6101 overwintering mosquitoes were collected, all of them females. Morphologically, the mosquitoes were assigned to five taxa: *An. maculipennis* complex (69 specimens, 21 pools), *Cs. annulata* (791 specimens, 162 pools), *Cx. hortensis/territans* (23 specimens, 8 pools), *Cx. pipiens* complex (5217 specimens, 530 pools), and *Uranotaenia unguiculata* (1 specimen, 1 pool) (Table S1).

### 3.2. Viral Screening

**Figure 1:**
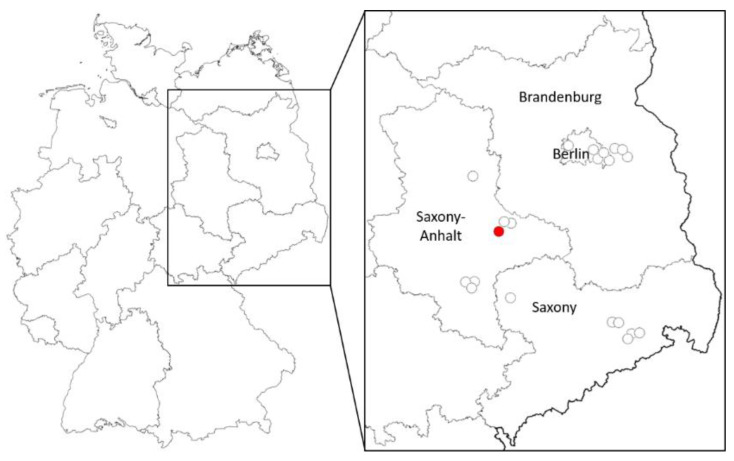
Locations in Germany with detected WNV overwintering (red dot) and of collection sites without virus detection (uncolored dots).

Among all the pools tested for viruses, one single pool collected on 9 March 2021 in a dungeon of a medieval castle in Rosslau, federal state of Brandenburg (Figure 1), was WNV-RNA-positive according to *NS5* and *NS2B-NS3* gene PCR and sequencing. Based on the number of mosquitoes tested, an infection prevalence of 0.016–0.20% was calculated, depending on the number of positive mosquitoes in the pool (1–12 positive specimens out of 6101 tested). The minimum infection rate (MIR) would thus be 0.16, assuming that only one mosquito included in the pool was positive [25].

Attempts to grow the virus from the respective mosquito homogenates on Vero and C3/36 cells remained unsuccessful, even after one blind passage, as checked by RT-qPCR. No full-length WNV-RNA could be extracted from the cells, whereas amplification and sequencing of the *NS2B-NS3* coding region of the RNA extracted from the original mosquito homogenate showed a complete match to the strain found in mosquitoes in 2019, which belonged to WNV lineage 2 [9,12].

### 3.3. Mosquito Identification

Mosquito species identification subsequent to virus detection suggested the WNV-RNA-positive pool comprised *Cx. pipiens* biotype *pipiens* specimens only.

## 4. Discussion

Mosquito taxa found in the hibernation shelters comprised species and species groups known to overwinter as females, and were therefore mainly as expected. However, an overwintering *Ur. unguiculata* female has never been encountered in Germany before, and its finding in Ihleburg, federal state of Saxony-Anhalt, adds to the particularly short list of only six collection sites for this species in Germany [26,27] (unpublished data).

Of the taxa collected, only members of the *Cx. pipiens* complex have been experimentally shown to be able to transmit WNV [28,29], although *An. maculipennis* complex species, *Cx. territans* and *Ur. unguiculata* have been found infected with WNV in the field [30,31,32,33]. In addition to *Cx. pipiens* complex species, several other European mosquito species are vector-competent for WNV [34], including species also overwintering in the female stage, such as *Cx. modestus* [35]. WNV-RNA, however, has so far only be demonstrated in overwintering *Cx. pipiens*.

In this study, WNV-RNA was found in only one mosquito pool, generating an MIR of 0.16 for all mosquitoes tested and of 0.19 for all *Cx. pipiens* specimens tested. The chance of demonstrating and isolating the virus from overwintering female mosquitoes can be increased by keeping still-living mosquitoes collected from their hibernation shelters at temperatures higher than 20 °C for some time (1–20 d) prior to examination [15,36], thus allowing the virus to restart replication. Hence, difficulties in recovering infectious virus from overwintering mosquitoes might, in part, be attributable to a lack of viral replication at low temperatures [36]. This implies that failure to isolate the virus directly from field-collected specimens in wintertime does not necessarily mean that the mosquitoes were not infected and unable to contribute to spreading the virus the following spring. For logistical reasons, in our study, mosquitoes could not be kept alive after collection, thereby severely limiting the chance of infectious virus isolation, and had to initially be stored at −20 °C instead of −80 °C, which would have been better for virus preservation.

MIRs similar to or slightly higher than in this study were obtained for overwintering *Cx. pipiens* in the Czech Republic (0.11 [16]) and in the U.S. (1.93 [13], 0.82 [14], and 0.04 [15], with the latter only considering *Cx. pipiens* biotype *pipiens*). In a more recent study from the Czech Republic, the MIR for hibernating *Cx. pipiens* reached an extraordinarily high value of 5.1, but in that case, only 198 mosquitoes were tested in four pools [17].

The MIRs calculated for WNV-RNA findings during mosquito activity periods, i.e., in the vegetative season, in areas of low to moderate endemicity, are mainly similarly low as in mosquito collections from resting places during wintertime, such as 0.78 for *Cx. pipiens* complex mosquitoes in a study carried out in Slovakia in 2018 and 2019 [37], or 1.05 in a study conducted in northern Italy in 2018 [38]. Further studies during the mosquito activity season did not find any mosquitoes positive for WNV-RNA at all, despite considerable numbers of specimens examined and diagnosed local/regional WNV cases in humans, birds and horses (e.g., [39,40]). On the other hand, MIRs for *Cx. pipiens* may be greater than 10 in highly endemic areas or during peak transmission periods, such as in New York state in 2000 [41]. In the Thessaly region of Greece, one of the European countries most affected by WNV, the MIR reached 2.6 in 2019 [42].

The infection prevalence reported here reflects the epidemiological situation of West Nile virus in Germany: annual case numbers are increasing, although still relatively low, and the first annual cases tend to occur sooner in the season (late summer in 2018 and 2019, and July in 2020) [43].

The identified positive mosquito species, *Cx. pipiens*, is considered the most important vector of WNV [2]. *Culex pipiens* consists of two biotypes or ecoforms, *pipiens* and *molestus*, which differ in their ecological characteristics. Among others, biotype *pipiens* preferentially feeds on birds, lives aboveground during the vegetative season, and undergoes winter diapause; biotype *molestus* is mainly mammalophilic, can often be found underground during the vegetative season, and remains active during wintertime [44,45]. Based on the overwintering behavior of the two biotypes, it is not surprising that the positive *Cx. pipiens* pool consisted of biotype *pipiens* only. Most of the *Cx. pipiens* specimens collected probably belonged to this biotype. In the only other study on WNV screening of overwintering mosquitoes that identified the *Cx. pipiens* biotypes, it was also *Cx. pipiens* biotype *pipiens* that was found to be infected [15]. Other studies demonstrating WNV in overwintering *Cx. pipiens* did not distinguish the biotypes [13,14,16,17].

The WNV strain found in the mosquito pool belonged to WNV lineage 2, and the sequence obtained from the two-step RT-PCR was identical to those found in positive mosquito pools collected in Germany in 2019, which could be expected as this was the predominant strain circulating in Germany in 2019 [9].

Although only WNV-RNA was detected, it is highly likely that the collected mosquito(es) had systemically become infected before its/their withdrawal into hibernation at the end of the 2020 mosquito season. First, *Cx. pipiens* biotype *pipiens* is a demonstrated WNV vector, i.e., the virus is able to replicate and disseminate in the mosquito. Second, as overwintering female mosquitoes rarely become active during wintertime to feed, virus uptake probably took place many weeks before, so that the virus would have been digested and its RNA completely degraded should the infection have been restricted to the mosquito midgut. Even in the rare case of the mosquito becoming active and feeding during wintertime, WNV infection during this occasion would be almost impossible due to a lack of infection sources at that time of the year. Although levels of infectious virus in overwintering mosquitoes would be low due to a lack of viral replication and slow degradation of virus particles and genomes, even very few full-length genomes in infected cells could restart replication with increasing temperatures and give rise to new infectious viral particles.

Although the persistence of WNV through the winter in vertebrates has been discussed, including oral infection of birds while scavenging at WNV-positive carcasses or of raptors while feeding on WNV-positive prey and bird-to-bird transmission within avian communities [46,47], the virus probably rather hides in overwintering mosquitoes, which can introduce it to naïve bird populations once temperatures rise in spring. Therefore, a region is likely to stay endemic once the virus has reached a certain level of circulation.

## Data Availability

Data are contained within the Supplementary Material.

## Supplementary Materials

The following are available online at https://www.mdpi.com/article/10.3390/v13122463/s1, Table S1: Mosquito collection and processing details.

## References

[1] Byas A.D., Ebel G.D. (2020). Comparative pathology of West Nile virus in humans and non-human animals. Pathogens.

[2] Bellini R., Zeller H., Van Bortel W. (2014). A review of the vector management methods to prevent and control outbreaks of West Nile virus infection and the challenge for Europe. Parasit. Vectors.

[3] Malkinson M., Banet C. (2002). The role of birds in the ecology of West Nile virus in Europe and Africa. Curr. Top. Microbiol. Immunol..

[4] Colpitts T.M., Conway M.J., Montgomery R.R., Fikrig E. (2012). West Nile virus: Biology, transmission, and human infection. Clin. Microbiol. Rev..

[5] Bowen R.A., Nemeth N.M. (2007). Experimental infections with West Nile virus. Curr. Opin. Infect. Dis..

[6] ECDC (European Center for Disease Prevention and Control) Epidemiological Update: West Nile Virus Transmission Season in Europe, 2018. https://www.ecdc.europa.eu/en/news-events/epidemiological-update-west-nile-virus-transmission-season-europe-2018.

[7] Camp J.V., Nowotny N. (2020). The knowns and unknowns of West Nile virus in Europe: What did we learn from the 2018 outbreak?. Expert Rev. Anti. Infect. Ther..

[8] Ziegler U., Lühken R., Keller M., Cadar D., Van der Grinten E., Michel F., Albrecht K., Eiden M., Rinder M., Lachmann L. (2019). West Nile virus epizootic in Germany, 2018. Antivir. Res..

[9] Ziegler U., Santos P.D., Groschup M.H., Hattendorf C., Eiden M., Höper D., Eisermann P., Keller M., Michel F., Klopfleisch R. (2020). West Nile virus epidemic in Germany triggered by epizootic emergence, 2019. Viruses.

[10] Pietsch C., Michalski D., Münch J., Petros S., Bergs S., Trawinski H., Lübbert C., Liebert U.G. (2020). Autochthonous West Nile virus infection outbreak in humans, Leipzig, Germany, August to September 2020. Euro Surveill..

[11] ECDC (European Center for Disease Prevention and Control) Distribution of West Nile Virus Infections among Human and Outbreaks among Equids and/or Birds in the EU, Transmission Season 2020. https://www.ecdc.europa.eu/sites/default/files/images/WNF_HumanAndAnimal_Historical2020.png.

[12] Kampen H., Holicki C.M., Ziegler U., Groschup M.H., Tews B.A., Werner D. (2020). West Nile virus mosquito vectors (Diptera: Culicidae) in Germany. Viruses.

[13] Nasci R.S., Savage H.M., White D.J., Miller J.R., Cropp B.C., Godsey M.S., Kerst A.J., Bennett P., Gottfried K., Lanciotti R.S. (2001). West Nile virus in overwintering *Culex* mosquitoes, New York City, 2000. Emerg. Infect. Dis..

[14] Bugbee L.M., Forte L.R. (2004). The discovery of West Nile virus in overwintering *Culex pipiens* (Diptera: Culicidae) mosquitoes in Lehigh County, Pennsylvania. J. Am. Mosq. Control Assoc..

[15] Farajollahi A., Crans W.J., Bryant P., Wolf B., Burkhalter K.L., Godsey M.S., Aspen S.E., Nasci R.S. (2005). Detection of West Nile viral RNA from overwintering pool of *Culex pipiens pipiens* (Diptera: Culicidae) in New Jersey. J. Med. Entomol..

[16] Rudolf I., Betášová L., Blažejová H., Venclíková K., Straková P., Šebesta O., Mendel J., Bakonyi T., Schaffner F., Nowotny N. (2017). West Nile virus in overwintering mosquitoes, Central Europe. Parasit. Vectors.

[17] Rudolf I., Šikutová S., Šebesta O., Mendel J., Malenovský I., Kampen H., Medlock J., Schaffner F. (2020). Overwintering of *Culex modestus* and other mosquito species in a reedbed ecosystem, including arbovirus findings. J. Am. Mosq. Control Assoc..

[18] Schaffner F., Angel G., Geoffroy B., Hervy J.P., Rhaiem A., Brunhes J. (2001). The Mosquitoes of Europe. An Identification and Training Programme.

[19] Becker N., Petric D., Zgomba M., Boase C., Dahl C., Madon M., Kaiser A. (2020). Mosquitoes.

[20] Heym E.C., Kampen H., Walther D. (2018). Mosquito species composition and phenology (Diptera, Culicidae) in two German zoological gardens imply different risks of mosquito-borne pathogen transmission. J. Vector Ecol..

[21] Vina-Rodriguez A., Sachse K., Ziegler U., Chaintoutis S.C., Keller M., Groschup M.H., Eiden M. (2017). A novel Pan-Flavivirus detection and identification assay based on RT-qPCR and microarray. Biomed. Res. Int..

[22] Eshoo M.W., Whitehouse C.A., Zoll S.T., Massire C., Pennella T.T., Blyn L.B., Sampath R., Hall T.A., Ecker J.A., Desai A. (2007). Direct broad-range detection of alphaviruses in mosquito extracts. Virology.

[23] Eiden M., Vina-Rodriguez A., Homann B., Ziegler U., Groschup M.H. (2010). Two new real-time quantitative reverse transcription polymerase chain reaction assays with unique target sites for the specific and sensitive detection of lineages 1 and 2 West Nile virus strains. J. Vet. Diagn. Investig..

[24] Grinev A., Daniel S., Stramer S., Rossmann S., Caglioti S., Rios M. (2008). Genetic variability of West Nile virus in US blood donors, 2002–2005. Emerg. Infect. Dis..

[25] CDC (Centers for Disease Control and Prevention) West Nile Virus—Resources. https://www.cdc.gov/westnile/resourcepages/mosqSurvSoft.html.

[26] Becker N., Kaiser A. (1995). Die Culicidenvorkommen in den Rheinauen des Oberrheingebiets mit besonderer Berücksichtigung von *Uranotaenia* (Culicidae, Diptera)—einer neuen Stechmückengattung für Deutschland. Mitt. Dtsch. Ges. Allg. Angew. Entomol..

[27] Tippelt L., Walther D., Kampen H. (2017). The thermophilic mosquito species *Uranotaenia unguiculata* Edwards, 1913 (Diptera: Culicidae) moves north in Germany. Parasitol. Res..

[28] Jansen S., Heitmann A., Lühken R., Leggewie M., Helms M., Badusche M., Rossini G., Schmidt-Chanasit J., Tannich E. (2019). *Culex torrentium*: A potent vector for the transmission of West Nile virus in central Europe. Viruses.

[29] Holicki C.M., Ziegler U., Răileanu C., Kampen H., Werner D., Schulz J., Silaghi C., Groschup M.H., Vasić A. (2020). West Nile virus lineage 2 vector competence of indigenous *Culex* and *Aedes* mosquitoes from Germany at temperate climate conditions. Viruses.

[30] Filipe A.R. (1972). Isolation in Portugal of West Nile virus from *Anopheles maculipennis* mosquitoes. Acta Virol..

[31] Lvov D.K., Butenko A.M., Gromashevsky V.L., Kovtunov A.I., Prilipov A.G., Kinney R., Aristova V.A., Dzharkenov A.F., Samokhvalov E.I., Savage H.M. (2004). West Nile virus and other zoonotic viruses in Russia: Examples of emerging-reemerging situations. Arch. Virol..

[32] CDC (Centers for Disease Control and Prevention) Mosquito Species in which West Nile Virus Has Been Detected, United States, 1999–2016. https://www.cdc.gov/westnile/resources/pdfs/MosquitoSpecies1999-2016.pdf.

[33] Kemenesi G., Dallos B., Oldal M., Kutas A., Földes F., Németh V., Reiter P., Bakonyi T., Bányai K., Jakab F. (2014). Putative novel lineage of West Nile virus in *Uranotaenia unguiculata* mosquito, Hungary. Virus Dis..

[34] Kampen H., Walther D. (2018). Vector potential of mosquito species (Diptera: Culicidae) occurring in Central Europe. Parasitol. Res. Monogr..

[35] Balenghien T., Vazeille M., Grandadam M., Schaffner F., Zeller H., Reiter P., Sabatier P., Fouque F., Bicout D.J. (2008). Vector competence of some French *Culex* and *Aedes* mosquitoes for West Nile virus. Vector-Borne Zoon. Dis..

[36] Dohm D.J., Turell M.J. (2001). Effect of incubation at overwintering temperatures on the replication of West Nile virus in New York *Culex pipiens* (Diptera: Culicidae). J. Med. Entomol..

[37] Čabanová V., Tichá E., Bradbury R.S., Zubriková D., Valentová D., Chovancová G., Grešáková L., Víchová B., Šikutová S., Csank T. (2021). Mosquito surveillance of West Nile and Usutu viruses in four territorial units of Slovakia and description of a confirmed autochthonous human case of West Nile fever, 2018 to 2019. Euro Surveill..

[38] Calzolari M., Angelini P., Bolzoni L., Bonilauri P., Cagarelli R., Canziani S., Cereda D., Cerioli M.P., Chiari M., Galletti G. (2020). Enhanced West Nile virus circulation in the Emilia-Romagna and Lombardy regions (northern Italy) in 2018 detected by entomological surveillance. Front. Vet. Sci..

[39] Klobucar A., Savic V., Curman Posavec M., Petrinic S., Kuhar U., Toplak I., Madic J., Vilibic-Cavlek T. (2021). Screening of mosquitoes for West Nile virus and Usutu virus in Croatia, 2015–2020. Trop. Med. Infect. Dis..

[40] Scaramozzino P., Carvelli A., Bruni G., Cappiello G., Censi F., Magliano A., Manna G., Ricci I., Rombolà P., Romiti F. (2021). West Nile and Usutu viruses co-circulation in Central Italy: Outcomes of the 2018 integrated surveillance. Parasit. Vectors.

[41] Bernard K.A., Maffei J.G., Jones S.A., Kauffman E.B., Ebel G., Dupuis A.P., Ngo K.A., Nicholas D.C., Young D.M., Shi P.Y. (2001). West Nile virus infection in birds and mosquitoes, New York State, 2000. Emerg. Infect. Dis..

[42] Papa A., Tsioka K., Gewehr S., Kalaitzopoulou S., Pappa S., Mourelatos S. (2020). West Nile virus lineage 2 in *Culex* mosquitoes in Thessaly, Greece, 2019. Acta Trop..

[43] FLI (Friedrich-Loeffler-Institut) West Nile Virus. https://www.fli.de/en/news/animal-disease-situation/west-nile-virus.

[44] Byrne K., Nichols R.A. (1999). *Culex pipiens* in London Underground tunnels: Differentiation between surface and subterranean populations. Heredity.

[45] Brugman V.A., Hernández-Triana L.M., Medlock J.M., Fooks A.R., Carpenter S., Johnson N. (2018). The role of *Culex pipiens* L. (Diptera: Culicidae) in virus transmission in Europe. Int. J. Environ. Res. Public Health.

[46] Montecino-Latorre D., Barker C.M. (2018). Overwintering of West Nile virus in a bird community with a communal crow roost. Sci. Rep..

[47] Reisen W.K., Wheeler S.S. (2019). Overwintering of West Nile virus in the United States. J. Med. Entomol..

